# Evaluating the impact of OMOP-CDM on data quality insight generation in respiratory disease management

**DOI:** 10.3389/fdata.2026.1744885

**Published:** 2026-04-10

**Authors:** Brenda Mbouamba Yankam, Fankoua Tchaptchet Luc Baudoin, Pauline Andeso, François Anicet Onana Akoa, Jean Blaise Ebimbe, Miranda Barasa, Mbele Onana, Samuel Iddi, Agnes Kiragga, Bertrand Hugo Mbatchou Ngahane, Data Science Without Borders Project

**Affiliations:** 1 Douala General Hospital, Data Science Without Borders Project, Douala, Cameroon; 2 Faculty of Mathematics, University of Bochum, Bochum, Germany; 3 African Population and Health Research Center, Nairobi, Kenya; 4 School of Health Sciences, Catholic University of Central Africa, Yaounde, Cameroon; 5 Douala Gynaeco-Obstetric and Pediatric Hospital, Douala, Cameroon; 6 Internal Medicine Department, Douala General Hospital, Douala, Cameroon; 7 Department of Statistics, University of Ghana, Accra, Ghana; 8 Infectious Diseases Institute, College of Health Sciences, Makerere University, Kampala, Uganda; 9 Faculty of Medicine and Pharmaceutical Sciences, University of Douala, Douala, Cameroon

**Keywords:** Achilles DQD, data quality, ETL process, OMOP CDM, respiratory diseases, standardization

## Abstract

The increasing volume and heterogeneity of patient care data present significant challenges for comprehensive analysis and the generation of insights, particularly in specific areas such as respiratory diseases. Standardizing diverse health data is crucial for enabling large-scale observational research and ensuring data readiness. The Observational Medical Outcomes Partnership (OMOP) Common Data Model (CDM) provides a widely adopted standard for harmonizing such data. However, evaluating the quality of data transformed into the OMOP CDM format is a critical step before its use in research or clinical decision support. This study evaluates the impact of the OMOP CDM standardization process on generating data quality insights for a respiratory disease dataset. The source dataset was initially paper-based, converted to an electronic format, and translated from French into English. This historical dataset covers the years 2009–2023 and contains 108 variables and 2,154 records. The data underwent the standard Extract, Transform, and Load (ETL) process to convert into the OMOP CDM format. Following this transformation, the quality of the resulting OMOP CDM instance was assessed. We utilized the Data Quality Dashboard (DQD) to evaluate the quality of the OMOP CDM database before and after ETL verification. DQD performs validation checks on the data based on key data quality dimensions, including completeness, plausibility, and conformance. Overall, the assessment conducted 2,344 checks, of which 2,269 passed, and 75 failed, resulting in a corrected pass rate of 96% for the Respiratory Diseases Inpatients data before ETL verification. After ETL verification, the assessment conducted 2,374 checks, of which 2,356 passed, and 40 failed, resulting in a 100% corrected pass rate. Standardizing respiratory disease data using the OMOP CDM enabled a structured and transparent evaluation of data quality. Through the application of the DQD, this study demonstrated the utility of OMOP CDM in generating meaningful data quality insights. These findings highlight the model's potential to enhance data readiness and support evidence-based decision-making in respiratory disease management.

## 1 Introduction

Observational health data, derived from sources like electronic health records (EHRs), claims, and registries, are increasingly available and hold significant potential for research and informing clinical practice (Blacketer et al., 2021). Using these data is crucial for understanding diseases and improving healthcare outcomes, particularly for respiratory diseases, HIV, tuberculosis, and stroke. However, these data sources are often disparate, featuring varying formats, structures, and terminologies (Biedermann et al., 2021; Papez et al., 2021). Standardizing such diverse data is a crucial prerequisite for enabling large-scale observational research and ensuring the data is ready for analysis and clinical decision support (Papez et al., 2021).

The Observational Medical Outcomes Partnership (OMOP) Common Data Model (CDM) has emerged as a widely adopted standard for harmonizing observational health data (Li and Tsui, 2020). The concept behind OMOP CDM is to transform data from various databases into a common format, allowing systematic analyses using standard analytic approaches (Li and Tsui, 2020). This approach aims to decrease challenges to data reuse and increase the likelihood that data will be used to answer scientific questions (Li and Tsui, 2020). The use of a standard terminology dictionary is a critical component of OMOP CDM, ensuring syntactic and semantic interoperability (Li and Tsui, 2020).

A critical step after transforming data into the OMOP CDM format is evaluating its quality. Concerns about the quality of observational health data are often voiced, especially as it is not primarily collected for research purposes (Blacketer et al., 2021). Different approaches have been taken to assess data quality in various databases (Blacketer et al., 2021). The Observational Health Data Sciences and Informatics (OHDSI) community, which develops and maintains the OMOP CDM (Wang et al., 2024) provides tools for data quality assessment. One such tool is the Data Quality Dashboard (DQD), which offers exhaustive quality checks of over 2,500 based on categories and contexts (Sathappan et al., 2021). It performs validation checks on conformance, completeness, and plausibility. The DQD also provides a dashboard for communicating and comparing data quality results (Sathappan et al., 2021). Studies have successfully used OHDSI's DQD in iterative processes to identify and fix data quality issues, thereby improving the quality and compliance of OMOP CDM databases (Blacketer et al., 2021; Marteau et al., 2024).

Evaluating data quality, particularly in the context of respiratory diseases following OMOP CDM standardization, is essential to ensure the reliability of subsequent research and decision-support systems. This study uses the DQD tool to evaluate the impact of the OMOP CDM standardization process on generating data quality insights for a respiratory disease dataset from Douala General Hospital (DGH).

## 2 Methods

### 2.1 Dataset description

The source dataset used in this study comprises a historical respiratory disease dataset. Initially available in a paper-based format (in French), which was converted to an electronic format: the digitization was performed with careful verification against original files to ensure accuracy, and translated from French into English using an automated translation approach implemented in R, followed by manual review by DGH clinicians to ensure semantic consistency that retains the actual meaning of the data prior to vocabulary mapping. To validate the semantic integrity of the translation, 90 of the 108 source variables and clinical terms were independently reviewed by two DGH clinicians familiar with the original French records. Each reviewer assessed whether the translated English term preserved the clinical meaning of the original French term. Discrepancies between the two reviewers were resolved through structured discussion and consensus. The inter-reviewer agreement rate was 94% (85 out of 90 terms agreed upon without revision), indicating a high level of semantic consistency between the translated and original French terms. The five terms with initial discrepancies were resolved by consensus prior to vocabulary mapping. For instance, atcd de pneumonie was translated as history of pneumonia, and asthme was translated as asthma, etc. The dataset was anonymized in accordance with data protection guidelines prior to upload to White Rabbit. White Rabbit provided a scan report that provided an overview of each variable, including its name, description, data type, proportion of missing values, and number of unique values. Comprehensive data cleaning, quality checks, and descriptive statistical analyses were performed before initiating the standardization pipeline, as shown in Table 1. Data cleaning was performed, including the removal of duplicates and the resolution of missing or inconsistent values by rechecking patient files. Missingness was generally low but varied by variable type: older paper records frequently lacked detailed symptom onset dates. Legibility issues in handwritten forms were resolved by cross-checking against the original patient files. This validation step, achieving a 94% inter-reviewer agreement rate across 90 of 108 variables, confirmed that automated translation did not alter the clinical interpretation of the original records prior to vocabulary mapping and OMOP standardization.

**Table 1:** Overview of source data characteristics identified via White Rabbit profiling.

Table	Field	Description	Type	Fraction empty	*N* unique values
respirdataset.csv	unique_code	unique_code	VARCHAR	0.0%	2,145
respirdataset.csv	Age	Age of the patient	REAL	0.0%	86
respirdataset.csv	Sex	Gender of the patient	VARCHAR	0.0%	2
respirdataset.csv	Origin service	Origin service	VARCHAR	0.0%	6
respirdataset.csv	Year of consultation	Year of consultation	INT	0.0%	15
respirdataset.csv	Payment method	Payment method	VARCHAR	0.0%	5
respirdataset.csv	Date hospitalized	Date hospitalized	DATE	0.0%	1,705
respirdataset.csv	Release date	Release date	VARCHAR	0.0%	1,612
respirdataset.csv	History of tuberculosis	History of tuberculosis	VARCHAR	0.0%	2
respirdataset.csv	location of your TB ant	location of your TB ant	VARCHAR	0.0%	3
respirdataset.csv	previous TB delay	previous TB delay (years)	VARCHAR	0.0%	2
respirdataset.csv	History of Asthma	History of Asthma	VARCHAR	0.0%	2
respirdataset.csv	History of GERD	History of GERD	VARCHAR	0.0%	2
respirdataset.csv	History of COPD	History of COPD	VARCHAR	0.0%	2
respirdataset.csv	History of Pneumonia	History of Pneumonia	VARCHAR	0.0%	2
respirdataset.csv	History of Diabetes	History of Diabetes	VARCHAR	0.0%	2
respirdataset.csv	History of CRF	History of chronic renal failure	VARCHAR	0.0%	2
respirdataset.csv	History of HTA	History of HTA	VARCHAR	0.0%	2
respirdataset.csv	History of Heart Disease	History of Heart Disease	VARCHAR	0.0%	2
respirdataset.csv	History of stroke	History of stroke	VARCHAR	0.0%	2
respirdataset.csv	History of viral hepatitis	History of viral hepatitis	VARCHAR	0.0%	2
respirdataset.csv	Type of hepatitis	Type of hepatitis	VARCHAR	0.0%	4
respirdataset.csv	Cancer History	Cancer History	VARCHAR	0.0%	2
respirdataset.csv	Cancer seat	The site where cancer is	VARCHAR	0.0%	8
respirdataset.csv	HIV status	HIV status before consultation	VARCHAR	0.0%	3
respirdataset.csv	Fever	Fever	VARCHAR	0.0%	2
respirdataset.csv	…	…	…	…	…
respirdataset.csv	Last CD4 delay in months	Last CD4 delay in months	VARCHAR	0.0%	40
respirdataset.csv	Preventive cotrimoxazole	Preventive cotrimoxazole	VARCHAR	0.0%	3
respirdataset.csv	ARV	Antiretroviral therapy	VARCHAR	0.0%	3

The dataset covered the period from 2009 to 2023 and comprised 108 variables and 2,154 records, reflecting all accessible cases documented at the hospital during this period. The variables in this dataset can be categorized as follows: demographics such as age and sex; clinical history such as history of pneumonia, history of asthma, History of chronic renal failure, and history of stroke; symptoms at presentation such as chest pain, cough, fever, dyspnea, anorexia, and night sweating; laboratory and diagnostic tests: leukocytes, lymphocytes, neutrophils, hemoglobin, and platelet counts; outcomes such as returned home, death, transfer to another hospital, intensive care unit, etc. No imaging or other complex data were included. Table 2 presents descriptive statistics for selected categories.

**Table 2:** Frequency and percentage of participants with a history of pneumonia, a history of asthma, and cough, stratified by age and sex.

Socio-demographic characteristics	History of pneumonia		History of asthma		Cough		Total
	Yes	No	Yes	No	Yes	No	
Age	*N* = 136	*N* = 2,018	*N* = 95	*N* = 2,059	*N* = 735	*N* = 1,419	*N* = 2,154
15–20	6 (4.41)	56 (2.78)	5 (5.26)	57 (2.77)	41 (5.58)	21 (1.48)	62 (2.9)
21–35	18 (13.24)	390 (19.33)	19 (20.00)	389 (18.89)	296 (40.27)	112 (7.89)	408 (18.9)
36–45	27 (19.85)	415 (20.56)	19 (20.00)	423 (20.54)	280 (38.10)	162 (11.42)	442 (20.5)
46–55	27 (19.85)	378 (18.73)	8 (8.42)	397 (19.28)	264 (35.92)	141 (9.94)	405 (18.8)
56–65	28 (20.59)	329 (16.30)	15 (15.79)	342 (16.61)	225 (30.61)	132 (9.30)	357 (16.6)
66–75	11 (8.09)	270 (13.38)	15 (15.79)	266 (12.92)	180 (24.49)	101 (7.12)	281 (13.0)
76–85	15 (11.03)	130 (6.44)	9 (9.47)	136 (6.61)	97 (13.20)	48 (3.38)	145 (6.7)
86–95	3 (2.21)	43 (2.13)	5 (5.26)	41 (1.99)	32 (4.35)	14 (0.99)	46 (2.1)
96 and above	1 (0.74)	7 (0.35)	0 (0.00)	8 (0.39)	4 (0.54)	4 (0.28)	8 (0.4)
Sex							
Female	63 (46.32)	920 (45.59)	63 (66.32)	920 (44.68)	349 (47.48)	634 (44.68)	983 (45.6)
Male	73 (53.68)	1,098 (54.41)	32 (33.68)	1,139 (55.32)	386 (52.52)	785 (55.32)	1,171 (54.4)

Among the 2,154 participants included in the analysis, 136 (6.3%) reported a history of pneumonia, 95 (4.4%) reported a history of asthma, and 735 (34.1%) reported having a cough. The age distribution showed that the largest proportion of participants was aged 36–45 years (20.5%), followed by 21–35 years (18.9%) and 46–55 years (18.8%). Histories of pneumonia and asthma were most frequently reported among individuals aged 36–65 years, while cough was more commonly reported in the 21–55 years age group, particularly among those aged 21–35 years (40.3%). Female participants accounted for 45.6% of the total sample, whereas male participants accounted for 54.4%. A slightly higher proportion of males reported a history of pneumonia (53.7%) and cough (52.5%), whereas a higher proportion of females reported a history of asthma (66.3%).

### 2.2 Data standardization to OMOP CDM

**Figure 1:**
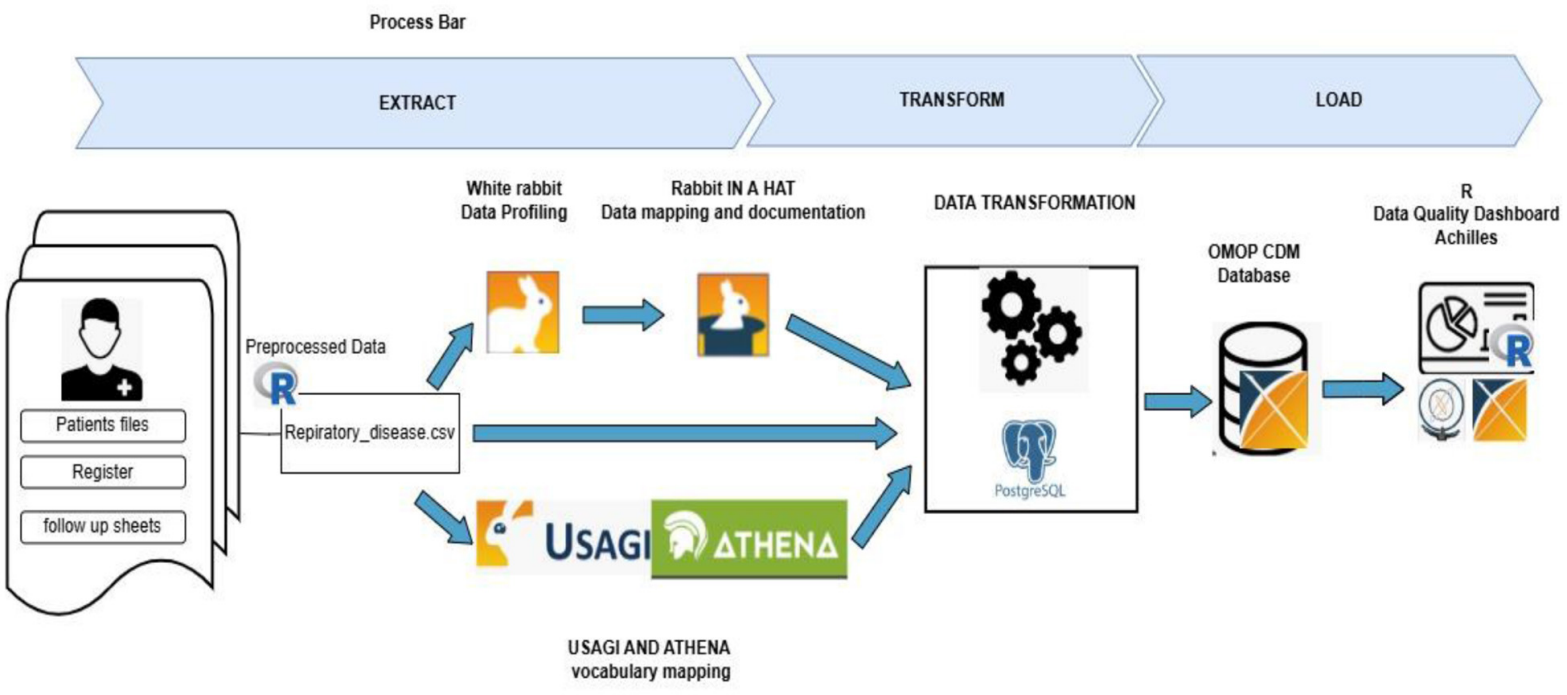
ETL pipeline for standardizing the respiratory disease dataset to the OMOP CDM.

The source data underwent the standard Extract, Transform, and Load (ETL) process to convert into the OMOP CDM format, as illustrated in Figure 1. According to OMOP guidelines, the ETL process for medical data comprises several key steps: mapping vocabularies, aligning source data tables with CDM tables, transforming and loading local data into the CDM structure, and validating the integrity and equivalence of the transformed data against the source data (Li and Tsui, 2020).

**Figure 2:**
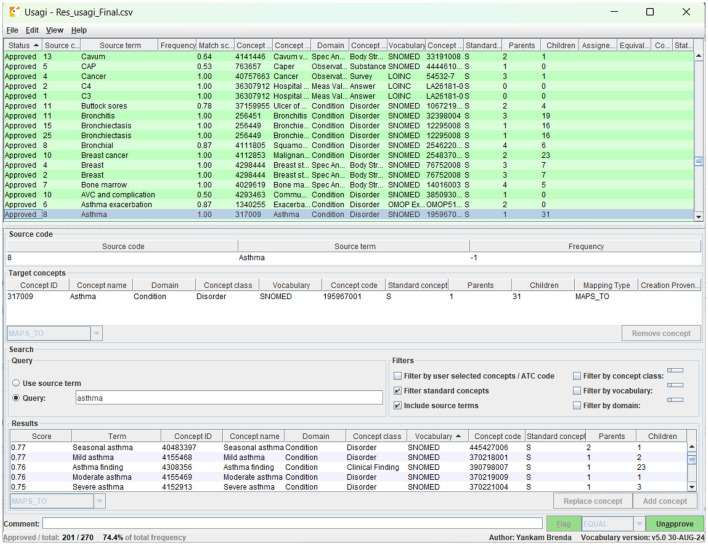
Concept mapping to OMOP standard vocabularies using Usagi.

Moreover, source concepts were mapped to OMOP standard vocabularies, such as SNOMED CT (Systematized Nomenclature of Medicine Clinical Terms), LOINC (Logical Observation Identifiers Names and Codes), and RxNorm (standardizes medications and drugs), using Usagi and Athena, as shown in Figure 2. The original French term from patient records, for instance, asthme, was translated to English as asthma during digitization.

The term was imported into Usagi, which generated several candidate concepts from the standard vocabulary, including concept ID: 317009 for asthma, concept ID: 4155468 for mild asthma, and concept ID: 4155469 for moderate asthma, as shown in the Results section of Figure 2. Figure 2 summarizes the Usagi vocabulary mapping workflow, demonstrating how source terms such as asthma and tuberculosis were evaluated and mapped to standard OMOP concepts, with candidate selection and final assignment reviewed by clinicians.

Based on clinical context and the source data, asthma in the patient records referred to any documented history of asthma, without specifying subtype. Candidate concepts were evaluated for semantic fit and clinical relevance. Terms with insufficient specificity, such as mild asthma, were excluded to preserve the original clinical concept of the data. Hence, the variable was mapped to concept ID 317009, which refers to asthma and is mapped as a condition, as highlighted in Figure 2, since it best represented the source term in a clinically accurate and standardized way. The mapping was reviewed by DGH clinicians and subsequently by the Data Science Without Borders Technical team.

**Figure 3:**
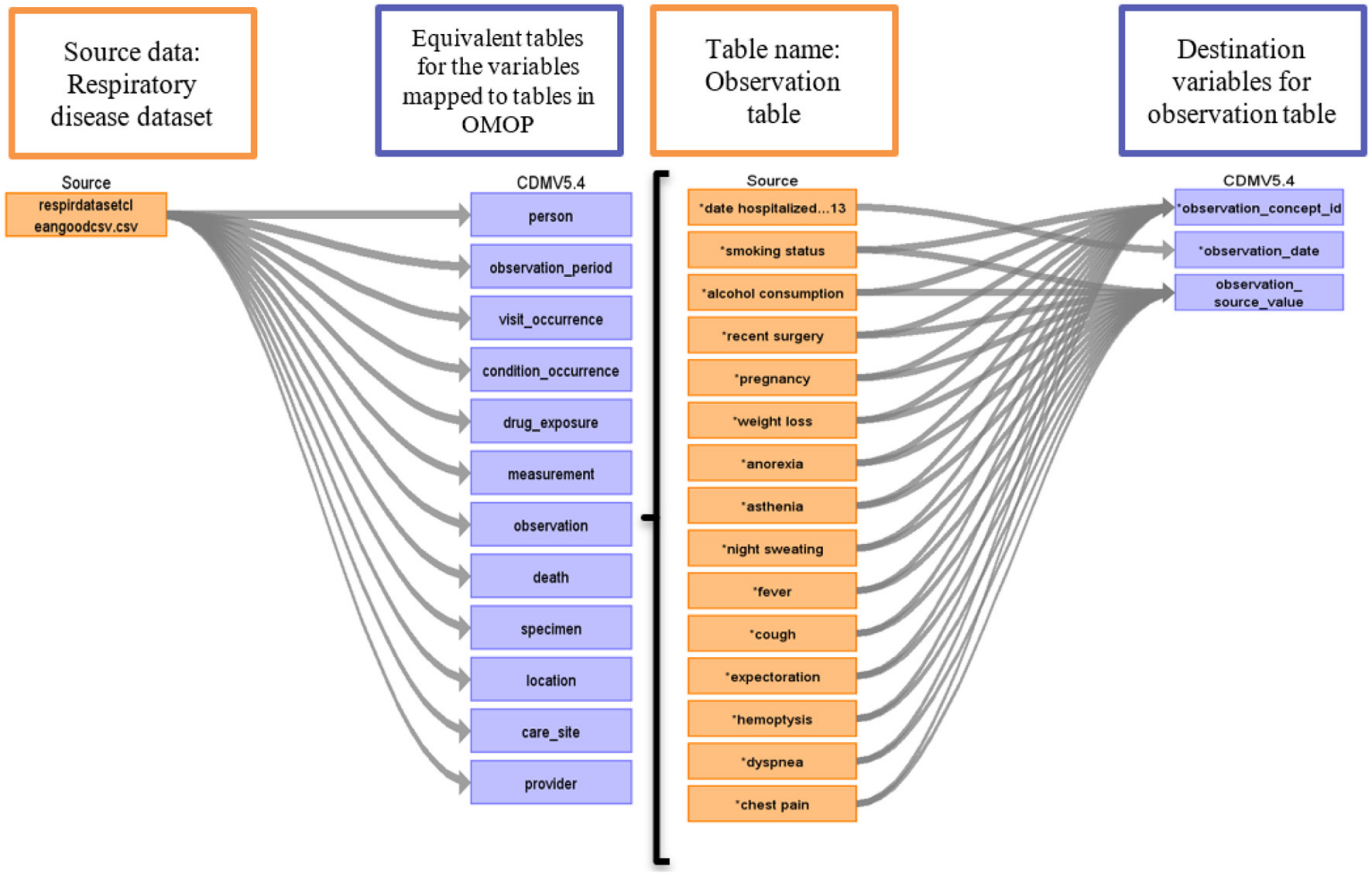
ETL mapping of respiratory disease source data to the OMOP CDM OBSERVATION Table.

Furthermore, Rabbit-In-A-Hat, a tool used to design and document the ETL, was used to define how source tables and columns align with OMOP CDM structures (Blacketer et al., 2021; Li and Tsui, 2020). This ETL process is illustrated in Figure 3 and summarized in Table 3. As a result of this process, the following OMOP CDM tables were populated: person, observation_period, visit_occurrence, condition_occurrence, drug_exposure, measurement, observation, death, specimen, location, care_site, and provider.

**Table 3:** ETL specification document extract from the OBSERVATION table with some variables.

Destination field	Source field	Comment
observation_id		The unique key given to an observation record for a Person: Autogenerated
person_id		# Foreign key to person Table
observation_concept_id	smoking status alcohol consumption recent surgery pregnancy weight loss anorexia asthenia night sweating fever cough expectoration hemoptysis dyspnea chest pain	# Indicate the concept ID for all the observations: smoking status, alcohol consumption, recent surgery, pregnancy...
observation_date	date hospitalized...13	
observation_datetime		
observation_type_concept_id		# Indicate the 38000280 EHR administration record
value_as_number		# Set to Null
value_as_string		# Indicate the observation levels
value_as_concept_id		# Indicate concept id for yes (4188539), and concept id for No =4188540
qualifier_concept_id		
unit_concept_id		
provider_id		
visit_occurrence_id		# Foreign key from Visits occurrence table.
visit_detail_id		
observation_source_value	smoking status alcohol consumption recent surgery pregnancy weight loss anorexia asthenia night sweating fever cough expectoration hemoptysis dyspnea chest pain	# Verbatim values that appear from the source data.
observation_source_concept_id		#set to Null
unit_source_value		#set to Null
qualifier_source_value		#set to Null
value_source_value		#set to Null
observation_event_id		#set to Null
obs_event_field_concept_id		#set to Null

Figure 3 presents the ETL mapping of a subset of the respiratory disease dataset to the relevant OMOP CDM tables, with a focus on the observation table. For instance, hospitalization data was mapped to the observation_date field, while smoking status was mapped to both observation_concept_id and observation_source_value. Other variables, such as alcohol consumption, recent surgery, pregnancy, weight loss, anorexia, asthenia, night sweats, fever, cough, expectoration, hemoptysis, dyspnea, and chest pain, were also mapped to the observation_concept_id and observation_source_value fields. Hence, Figure 3 visually represents how these clinical concepts from the source data were integrated into the OMOP CDM's observation table. The OMOP CDM is supported by a standardized terminology system comprising 81 vocabularies, which ensures both syntactic and semantic interoperability (Li and Tsui, 2020).

During the ETL process, source data were assigned to OMOP CDM domains based on their clinical meaning and OMOP domain conventions. The source dataset did not contain standardized clinical codes such as SNOMED CT, LOINC, or RxNorm. As a result, domain assignment could not be automated through direct code-to-domain mapping. Instead, vocabulary mapping and domain selection were performed manually using expert judgment and OMOP CDM guidance, such that, condition domain was used for documented diagnoses and disease states such as history of tuberculosis, history of asthma, history of pneumonia etc; measurement domain was used for quantitative laboratory results such as neutrophils, leukocytes, smoking index, hemoglobin, etc; observation domain was used for clinical findings, symptoms, and descriptive attributes such as fever, cough, chest pain, dyspnea etc; person domain was used to represent individual patients and to store core demographic information such as sex, gender, year of consultation, race, and ethnicity.

Source values were mapped to standard OMOP concepts where possible by manually selecting appropriate standard concept IDs from the OMOP vocabulary.

However, during the mapping process, several challenges were encountered, particularly in mapping local professions in Cameroon, such as specific nurses or technicians who lacked predefined codes in OMOP's standard vocabulary; consequently, no solution was found, leaving a gap in the standardization of these professions in the dataset. Also, the inability to map terms, such as failure, new cases, and chylous, which were not found in Usagi and Athena. However, these terms were documented.

Another issue was that several laboratory measurements, such as creatinine and respiratory rate, lacked standard units, leading to inconsistencies. However, consultation with a domain expert from the Data Science Without Borders Technical team was done to standardize measurement units in accordance with medical conventions.

Table 3 presents a corresponding section of the ETL specification document, highlighting selected variables included in this ETL mapping. Access to the script that was used can be found https://github.com/aphrc-dswb/Douala-General-Hospital-DSWB, and access to the dataset can be obtained upon request.

An expert review of the entire process was conducted by the Data Science Without Borders (DSWB) technical team. Finally, the ETL process was implemented in PostgreSQL to transform and load data into OMOP CDM-compliant tables, including condition_occurrence, observation, care_site, and others (Blacketer et al., 2021; Li and Tsui, 2020; Sathappan et al., 2021). The PostgreSQL server connection was established using an R script.

### 2.3 Data quality assessment

Following the transformation to the OMOP CDM format, the quality of the resulting data instance was assessed. The study utilized the DQD tool, part of the OHDSI suite, to evaluate the quality of the OMOP CDM database for respiratory disease. DQD performs validation checks based on key data quality dimensions: conformance (adherence to standards), completeness (presence of values), and plausibility (believability of values) (Sathappan et al., 2021).

Failed checks were reviewed in a structured and iterative manner by examining the DQD output reports to identify the affected tables, fields, concepts, and violation types. Each failure was traced back to its likely root cause by inspecting the corresponding ETL logic, source-to-target mappings, and underlying source data. Remediation actions included correcting ETL transformation rules, such as data type conversions; refining vocabulary mappings to standard OMOP concepts; and adjusting join logic and constraints to ensure referential integrity.

Following these targeted ETL revisions, the OMOP CDM instance was regenerated, and the DQD was rerun to confirm whether previously failed checks were resolved. This iterative review, remediation, and verification cycle enabled systematic improvement of plausibility and conformance checks, while completeness-related failures were largely attributable to limitations in the source data and therefore remained unchanged.

## 3 Results

This section presents insights into data quality generated using the OMOP CDM and DQD.

### 3.1 Overall data quality performance

**Figure 4:**
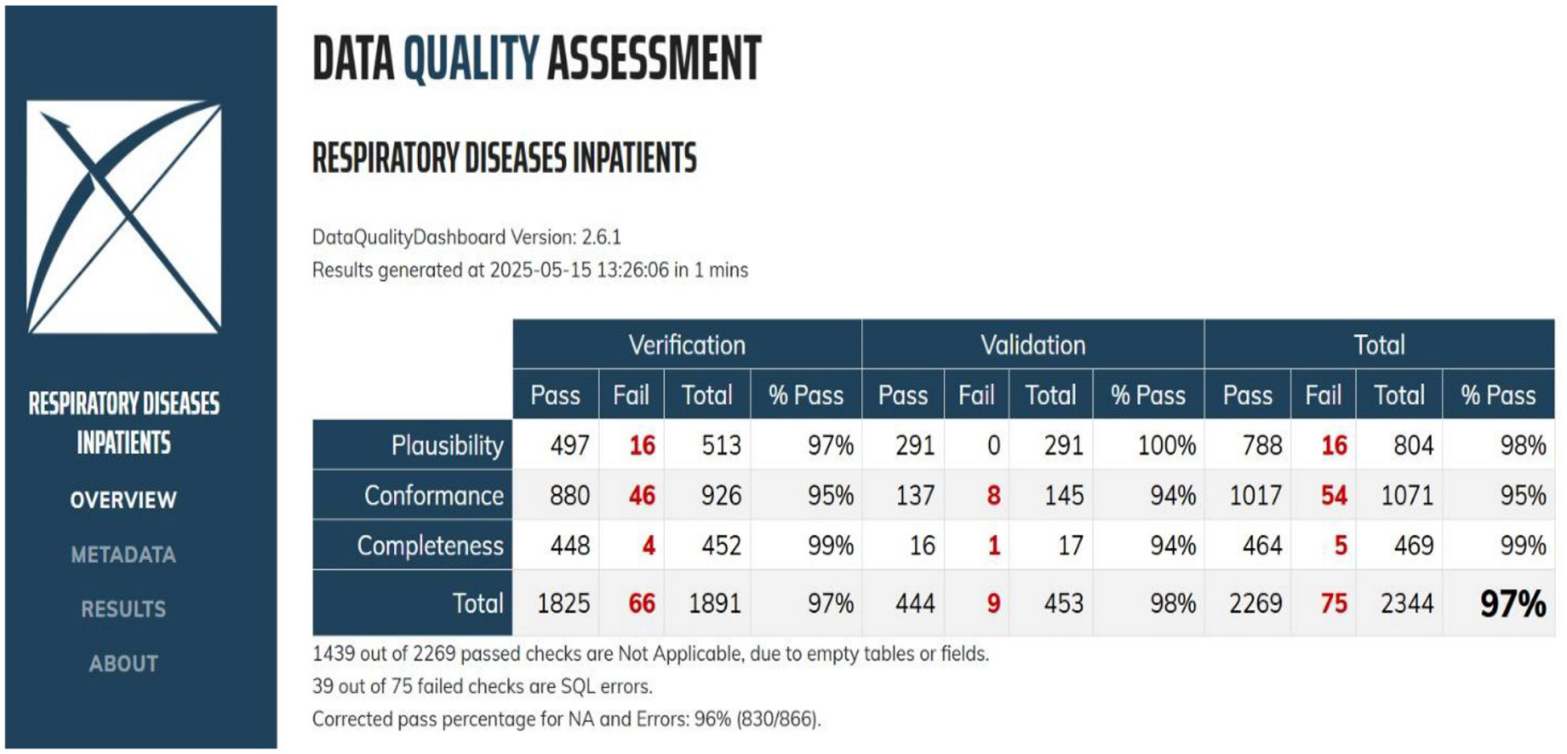
Data quality assessment of the respiratory disease dataset.

The assessment of the respiratory disease dataset, after its transformation into the OMOP CDM format, shows a high overall data quality. A total of 2,344 checks were conducted, with 2,269 passing and 75 failing, yielding a 96% corrected pass rate for the respiratory disease inpatient data, as shown in Figure 4. This indicates that the OMOP CDM standardization process successfully generated detailed insights into the dataset's quality across conformance, completeness, and plausibility dimensions.

Completeness checks, which ensure the presence of data values, performed well, with an overall pass of 454 out of 469, leading to a 99% pass rate.

Plausibility checks demonstrated strong performance, with an overall pass of 788 out of 884, leading to a 98% pass rate. Conformance checks, which evaluate whether data adheres to specified standards and formats, show that 1,071 out of 1,017 pass. This culminated in an overall conformance pass rate of 95%, which indicates that the transformed data largely complies with the OMOP CDM structure and format.

### 3.2 Data quality performance after ETL verification of failed checks

**Figure 5:**
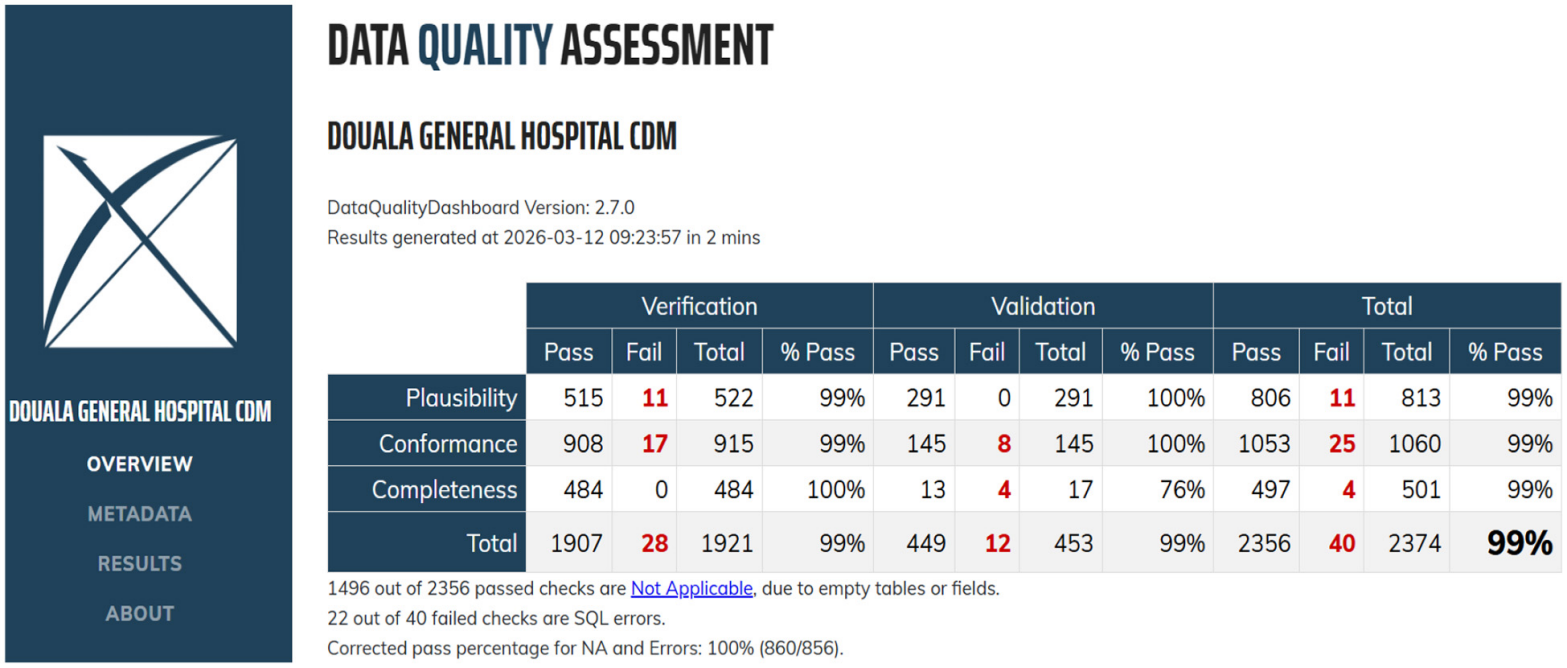
Data quality assessment after ETL verification of failed checks.

The results demonstrate a clear improvement in data quality following ETL verification of failed checks. Overall, the corrected pass rate increased from 96% before ETL verification to 100% after ETL verification, accompanied by a reduction in failed checks from 75 to 40, as presented in Figures 4, 5, respectively. This represents a substantial improvement in data quality and indicates that the ETL verification process effectively identified and resolved data issues. Improvements were observed primarily in the plausibility and conformance dimensions. Plausibility failures decreased from 16 to 11, resulting in an increase in the pass rate from 98% to 99%. This suggests enhanced logical consistency of the data after ETL processing, likely due to improved validation rules and correction of implausible values.

Conformance exhibited the most pronounced improvement, with failed checks reduced from 54 to 25 and the pass rate increasing from 95% to 99%. Also, completeness changed slightly from 5 to 4 failed checks and a pass rate of 99% reported before and after ETL verification. This suggests that missing data issues are largely attributable to upstream data generation or collection processes and are not readily resolved through ETL verification alone.

Overall, the ETL verification process resulted in a 46.7% reduction in total data quality failures, underscoring its critical role in the data processing pipeline. While ETL verification substantially improves plausibility and conformance, further improvements in completeness may require interventions at the data source level.

### 3.3 Analysis of removed tests before and after ETL verification of failed checks

Table 4, which details tests removed due to missing tables and columns, offers further insights into data structural limitations. The CONCEPT_plausibleGender test, categorized under Plausibility, was 100% removed (287 out of 287 total tests), suggesting either a complete absence of gender-related concepts in the mapped data or issues with how gender data were handled during the ETL process (Kahn et al., 2016). Similarly, FIELD_fkClass (Conformance category) and FIELD_sourceConceptRecordCompleteness (Completeness category) were 100% removed, though for a smaller number of tests (3 and 13, respectively). These removals highlight specific areas in which the original dataset's structure did not align with the expectations of the OMOP CDM or in which source concepts could not be fully mapped, resulting in unrunnable tests.

**Table 4:** Tests removed due to missing tables and columns in the OMOP CDM database before ETL verification.

Scripts names	# removed tests	% removed test	# total test	Category
FIELD_fkClass	3	100%	3	Conformance
CONCEPT_plausibleGender	287	100%	287	Plausibility
FIELD_plausibleGenderUseDescendants	4	100%	4	Plausibility
FIELD_sourceConceptRecordCompleteness	13	100%	13	Completeness
CONCEPT_plausibleUnitConceptIds	172	95%	181	Plausibility
FIELD_plausibleAfterBirth	39	80%	49	Plausibility
FIELD_plausibleBeforeDeath	37	79%	47	Plausibility
FIELD_plausibleTemporalAfter	40	78%	51	Plausibility
FIELD_plausibleValueLow	53	78%	68	Plausibility
FIELD_plausibleStartBeforeEnd	17	77%	22	Plausibility
FIELD_plausibleDuringLife	26	76%	34	Plausibility
FIELD_plausibleValueHigh	46	75%	61	Plausibility
FIELD_isForeignKey	121	74%	163	Conformance
FIELD_cdmDatatype	144	73%	197	Conformance
FIELD_isStandardValidConcept	45	70%	64	Conformance
FIELD_standardConceptRecordCompleteness	32	67%	48	Completeness
FIELD_fkDomain	29	64%	45	Conformance
TABLE_measurePersonCompleteness	10	63%	16	Completeness
FIELD_isRequired	90	62%	145	Conformance
FIELD_sourceValueCompleteness	19	59%	32	Completeness
FIELD_isPrimaryKey	13	57%	23	Conformance
FIELD_withinVisitDates	5	56%	9	Conformance
FIELD_measureValueCompleteness	194	54%	359	Completeness
FIELD_cdmField	0	0%	391	Conformance
TABLE_cdmTable	0	0%	31	Conformance
TABLE_measureConditionEraCompleteness	0	0%	1	Completeness

The validation tests are highlighted in blue and the verification tests in green.

Following ETL verification, Table 5 demonstrates both improvements and persistent challenges. The most notable improvement is observed in FIELD_cdmField, which dropped from 0% removed (all 391 tests runnable) before ETL to only 3% removed (11/391) after, indicating that ETL corrections substantially resolved field-level structural gaps. TABLE_measurePersonCompleteness also improved markedly, falling from 63% to 0% removed, suggesting successful resolution of person-level completeness mapping issues.

**Table 5:** Tests removed due to missing tables and columns in the OMOP CDM database after ETL verification.

Scripts names	# removed tests	% removed test	# total test	Category
cdmDatatype	140	71%	197	Conformance
cdmField	11	3%	391	Conformance
cdmTable	0	0%	31	Conformance
fkClass	2	67%	3	Conformance
fkDomain	27	63%	43	Conformance
isForeignKey	119	73%	163	Conformance
isPrimaryKey	13	57%	23	Conformance
isRequired	87	60%	145	Conformance
isStandardValidConcept	46	72%	64	Conformance
measureConditionEraCompleteness	0	0%	1	Completeness
measureObservationPeriodOverlap	0	0%	1	Plausibility
measurePersonCompleteness	0	0%	16	Completeness
measureValueCompleteness	231	59%	391	Completeness
plausibleAfterBirth	39	80%	49	Plausibility
plausibleBeforeDeath	37	79%	47	Plausibility
plausibleDuringLife	27	79%	34	Plausibility
plausibleGender	287	100%	287	Plausibility
plausibleGenderUseDescendants	4	100%	4	Plausibility
plausibleStartBeforeEnd	16	73%	22	Plausibility
plausibleTemporalAfter	39	76%	51	Plausibility
plausibleUnitConceptIds	176	97%	181	Plausibility
plausibleValueHigh	45	75%	60	Plausibility
plausibleValueLow	52	76%	68	Plausibility
sourceConceptRecordCompleteness	13	100%	13	Completeness
sourceValueCompleteness	24	75%	32	Completeness
standardConceptRecordCompleteness	33	69%	48	Completeness
withinVisitDates	6	67%	9	Plausibility

However, several checks showed little to no improvement or even slight increases in removal rates. CONCEPT_plausibleGender remained at 100% removal (287/287), confirming that gender-related mapping issues were not addressed during ETL verification. The Sex variable (Male/Female) is fully complete across all 2,154 records (0% missingness, Table 1). Rather, this reflects a specific ETL mapping gap: during the ETL process, the source gender values were not resolved to their corresponding standard OMOP gender concept IDs (concept ID 8507 for Male and 8532 for Female from the OMOP Gender vocabulary). As a result, the gender_concept_id field in the OMOP CDM person table was not populated with valid standard concepts, rendering all 287 DQD plausibility checks unrunnable. As shown in Table 5, this issue persisted after ETL verification, as the remediation cycle in this iteration prioritized conformance and structural corrections. CONCEPT_plausibleUnitConceptIds increased marginally from 95% to 97%, and FIELD_sourceValueCompleteness rose from 59% to 75%, suggesting that ETL corrections may have exposed additional unmapped source values rather than resolving them. Most remaining checks, including FIELD_cdmDatatype (73% to 71%), FIELD_isForeignKey (74% to 73%), and FIELD_isRequired (62% to 60%), showed only marginal reductions, reflecting limited structural gains across Conformance checks overall.

## 4 Discussion

This section discusses the impact of OMOP CDM on data quality insight generation and compares it with other studies.

### 4.1 The impact of OMOP CDM on data quality insight generation

This study successfully demonstrates that standardizing a respiratory disease dataset to the OMOP CDM significantly improves data quality insights. The application of the DQD facilitated a structured and transparent evaluation across the critical data quality dimensions of conformance, completeness, and plausibility. The overall corrected pass rate of 96% and 100% before and after ETL verification highlights the usefulness of OMOP CDM in preparing varied health data for large-scale observational research and evidence-based decision-making in respiratory disease management, consistent with the foundational goals of the OHDSI community (Hripcsak et al., 2015).

The ability of the OMOP CDM to clearly identify detailed data quality problems is consistent with previous studies showing that common data models support systematic, repeatable, and comparable data quality assessments across different institutions and settings (Kahn et al., 2016; Blacketer et al., 2021).

The high pass rates for plausibility (98% and 99%) and completeness (99%), before and after ETL verification, respectively, indicate that the transformed data is generally accurate and follows the prescribed OMOP CDM format. While conformance checks performed well (95% and 99% before and after ETL verification, respectively), the detailed results, particularly the instances of deleted tests due to missing tables and columns (Table 4), reflect common issues faced during OMOP CDM implementation. Similar limitations have been reported in multiple OMOP conversion efforts, where source systems lack certain clinical domains or represent them in non-standard ways (Sathappan et al., 2021; Quiroz et al., 2021; Maier et al., 2018).

For example, the complete removal of CONCEPT_plausibleGender before ETL verification suggests that certain expected data elements or their corresponding concepts were either absent in the source data or could not be mapped according to OMOP CDM conventions (Blacketer, 2024). Such findings highlight the importance of early data profiling and iterative ETL refinement, as recommended in established OMOP ETL frameworks (Quiroz et al., 2021).

Furthermore, the observed stability of completeness checks before and after ETL verification suggests that missingness is primarily driven by upstream data capture practices rather than transformation errors. This observation echoes findings from population health and registry-based OMOP implementations, where improvements in completeness often require changes at the data collection or documentation level rather than post hoc ETL adjustments (Ochola et al., 2025; Kiwuwa-Muyingo et al., 2023).

Importantly, the 46.7% reduction in total data quality failures following ETL verification represents the value of an iterative, DQD guided remediation cycle. The pronounced improvement in conformance checks, that is, from 95% to 99% pass rate, demonstrates that many structural misalignments identified during the initial DQD run were addressable through targeted ETL corrections, such as refining data type conversions and adjusting referential integrity constraints. By contrast, the near-stable completeness pass rate (99% before and after ETL verification) reinforces the understanding that missingness is largely a product of upstream data capture limitations inherent to paper-based and historically digitized records, rather than a consequence of the transformation process itself. This distinction is practically important: it directs future quality improvement efforts toward the point of data collection rather than the ETL pipeline, and it aligns with the Kahn et al. (2016) data quality framework, which differentiates between data quality issues arising from source system limitations vs. those introduced during data transformation.

### 4.2 Comparison with other studies

The findings of this study align with and build upon experiences from various OMOP CDM implementations across different healthcare systems and research networks. The study by Marteau et al. (2024), which utilized OHDSI's DQD, reported an overall pass rate of 83.2% from 1,125 tests. In comparison, our study reported a 96% corrected pass rate across 2,344 checks, suggesting either a cleaner initial dataset or a more mature ETL process for the respiratory disease data.

Similar to our findings, Marteau et al. (2024), encountered significant challenges with missing tables and columns, partly attributed to using an earlier OMOP CDM version. This resulted in the removal of many checks, 401 tests, and 1,718 rows, and affected multiple domains. Notably, issues such as CONCEPT_plausibleGender, which led to 100% removal in our analysis, were also present in this study (Marteau et al., 2024). The study by Marteau et al. (2024), also noted high percentages of violated rows for isForeignKey and measureValueCompleteness (Marteau et al., 2024), which are common challenges related to data conformance.

Furthermore, a study conducted by Blacketer et al. (2021), involving 15 data partners across the European health data and evidence network, demonstrated how the DQD iteratively improved data quality, particularly excelling at identifying and fixing conformance issues (Blacketer et al., 2021). While this study observed less dramatic improvements in completeness and plausibility checks (Blacketer et al., 2021), our study's high pass rates across all three categories after ETL verification, including 99% for completeness and 99% for plausibility, and 99% for conformance, suggest a strong underlying data quality. Blacketer et al. (2021) highlighted that the more proprietary source codes were mapped to standard concepts, the more checks the DQD could leverage, indicating that extensive vocabulary mapping directly contributes to a more comprehensive quality assessment. The large number of checks performed in our study of 2,374, compared to the median of 883 total checks in Blacketer et al. (2021) further supports the comprehensiveness of our evaluation.

A feasibility study involving Singaporean EHR and questionnaire data provides a particularly relevant comparison (Sathappan et al., 2021). This study, which also used the DQD, reported that 38 out of 2,622 checks failed (1.4%), which is very close to our study's 75 failed checks out of 2,344 (approximately 3.2%) before ETL verification. Moreover, there was found to be better content coverage of over 90% for clinical data, but significantly lower coverage of around 11% for questionnaire data (Sathappan et al., 2021). This aligns with challenges identified in our study, such as the difficulty in mapping past medical histories or local professions lacking predefined OMOP codes. This study notes that challenges remain for standardizing cognitive and depression assessment questionnaire data, reinforcing this common hurdle (Sathappan et al., 2021).

Beyond single-institution studies, broader reviews further contextualize our findings. A scoping review on OMOP CDM adoption for cancer research by Wang et al. (2024), identified data quality as a significant challenge, encompassing issues like poor record linkage, data timeliness, and limitations in OMOP vocabularies. The study by Wang et al. (2024) indicates that a substantial portion of the reviewed studies in the infrastructure theme did not perform mapping quality evaluation. This contrasts with the comprehensive DQD evaluation in our study, which is in contrast to the comprehensive DQD-based assessment performed in our work. This highlights the added value of embedding systematic data quality evaluation in OMOP CDM implementations, particularly for disease-focused datasets.

From a global health perspective, OMOP CDM implementations in African and low- and middle-income country (LMIC) contexts remain limited but are rapidly expanding (Ochola et al., 2025; Kiwuwa-Muyingo et al., 2023; Bhattacharjee et al., 2024). Studies demonstrating the harmonization of population health and infectious disease data into OMOP, including COVID-19 serosurveillance and longitudinal demographic surveillance systems, report similar challenges related to incomplete domains and context-specific variables (Ochola et al., 2025; Bhattacharjee et al., 2024). Given the high burden of respiratory diseases, including tuberculosis, in sub-Saharan Africa (Zumla et al., 2015), the findings of this study provide important methodological evidence supporting the feasibility of applying OMOP CDM and OHDSI tools in such settings.

Overall, this study strengthens existing evidence that integrating OMOP CDM with robust ETL verification and DQD supports detailed and actionable data quality evaluation. It reinforces prior findings that while standardization improves transparency and analytical readiness, achieving optimal data quality remains an iterative process that depends on source system characteristics, vocabulary coverage, and sustained investment in data governance (Hripcsak et al., 2015; Kahn et al., 2016; Voss et al., 2024).

Notably, this study extends the evidence base to a sub-Saharan African clinical setting, where OMOP CDM implementations remain relatively sparse compared to high-income country contexts. The challenges encountered here, including the absence of pre-existing standardized clinical codes, the need for manual vocabulary mapping via Usagi, and gaps in OMOP's standard vocabulary for local professions and context-specific clinical terms, are likely representative of broader obstacles facing LMIC institutions seeking to adopt OMOP CDM. Unlike many published OMOP implementations that begin with EHR data already containing structured codes, this study demonstrates that the framework remains applicable even when source data originate from paper-based registries and require digitization and linguistic translation prior to ETL. The successful achievement of a 96% corrected pass rate under these conditions, rising to 100% after ETL verification, suggests that the OMOP CDM and DQD toolset is sufficiently robust and flexible to support high-quality data standardization in resource-constrained and historically under-represented health data environments.

### 4.3 Limitations of the study

Some of the limitations encountered during the process were:

1. The dataset comprised only 2,154 patient records spanning 15 years (2009–2023), equating to an average of approximately 143 records per year. The absence of device, procedure, and unstructured data constrained the range of OMOP CDM tables populated. While this dataset reflects the complete set of accessible inpatient cases documented at DGH during this period, this level of annual record density introduces important constraints for temporal analysis. Specifically, year-level strata with ~143 observations may lack the statistical power required to reliably detect gradual trends in disease presentation, seasonal variation, or shifts in treatment practices over time. Sub-group analyses stratified simultaneously by year, age group, and sex are particularly susceptible to small-cell instability, which can produce unstable estimates and widen confidence intervals to the point of clinical uninterpretability. Furthermore, the uneven distribution of records across years, a common feature of historical paper-based registries, means that some years may be over- or under-represented, potentially introducing temporal bias in trend analyses. These constraints do not undermine the primary purpose of this study, namely, demonstrating the feasibility of OMOP CDM standardization and systematic DQD-based data quality assessment in a sub-Saharan African hospital setting. However, they should be carefully considered before using the OMOP-CDM instance for downstream longitudinal or predictive modeling.
2. Certain local healthcare roles in Cameroon, such as specific categories of nurses or technicians, did not have corresponding standardized codes within OMOP's vocabulary, resulting in potential loss of granularity in professional role data. Additionally, some locally used clinical terms, such as failure, new cases, and chylous, could not be mapped to standard OMOP concepts because they were not available in Usagi or Athena, even though they were documented in the source data.
3. Some laboratory and clinical measurements, including creatinine and respiratory rate, were recorded using non-standard units, leading to inconsistencies; these were addressed through consultation with a domain expert from the Data Science Without Borders Technical team to standardize units according to medical conventions. Although manual review helped mitigate risks, some information loss during digitization and linguistic translation may have occurred.
4. The dataset originates from Douala General Hospital, reflecting local clinical practices, documentation standards, and patient demographics. As a result, findings related to data structure, completeness, and mapping challenges may not be fully generalizable to other institutions or healthcare systems, particularly those with native electronic health records.

### 4.4 Research application

Some areas of application using this dataset include:

1. The OMOP-CDM representation supports systematic characterization of respiratory disease patients, including socio-demographic, symptom patterns, treatments, and outcomes, enabling consistent cohort definition and descriptive analyses using OHDSI tools.
2. Standardized condition and observation tables facilitate the investigation of associations between respiratory diseases and comorbid conditions such as asthma, diabetes, and hypertension, supporting epidemiological and hypothesis-generating studies.
3. The longitudinal structure of the OMOP-CDM enables analysis of temporal trends in disease presentation, treatment practices, and outcomes over the 15-year study period, allowing assessment of changes in clinical patterns and healthcare utilization.
4. The harmonization and quality-controlled dataset provides a suitable foundation for developing predictive models, for example, risk of hospitalization or adverse outcomes, while ensuring feature consistency and reproducibility.

## 5 Conclusion

The successful standardization of the respiratory disease dataset to the OMOP CDM and a rigorous data quality assessment using DQD have yielded valuable insights into the data's readiness for research. This study contributes to the growing evidence that OMOP CDM can effectively harmonies diverse health data, enable structured data quality evaluation, and support more trustworthy decision-making for patient care and research outcomes. The workflow and pipeline developed are adaptable and can be extended to other healthcare systems and disease areas, provided that careful attention is paid to the nuances of source data and the continuous refinement of mapping strategies.

## Data Availability

The raw data supporting the conclusions of this article will be made available by the authors, without undue reservation.
